# Secondary intrascleral intraocular lens (IOL) fixation with capsule preservation for IOL dislocation following mature cataract surgery with incomplete capsulorhexis: A case report

**DOI:** 10.1097/MD.0000000000043030

**Published:** 2025-06-20

**Authors:** Suguru Nakagawa, Kiyoshi Ishii

**Affiliations:** a Department of Ophthalmology, Saitama Red Cross Hospital, Saitama, Japan; b Department of Ophthalmology, Saitama Medical Center, Jichi Medical University, Saitama, Japan.

**Keywords:** cataract surgery, incomplete continuous curvilinear capsulorhexis, intrascleral intraocular lens fixation, iris fluttering, lens capsule preservation, pupillary intraocular lens capture

## Abstract

**Rationale:**

The objective of this study was to apply secondary intraocular lens (IOL) intrascleral fixation with lens capsule preservation in a patient with IOL dislocation following mature cataract surgery with incomplete continuous curvilinear capsulorhexis (CCC).

**Patient concerns:**

A 56-year-old Japanese woman experienced distorted vision 4 days after phacoemulsification and intracapsular IOL implantation for a mature cataract.

**Diagnoses:**

Slit-lamp examination revealed inferior-nasal dislocation of the intracapsular IOL through an anterior capsule defect (3–6 o’clock) caused by peripheral extension of the CCC during the primary surgery.

**Interventions:**

IOL extraction followed by intrascleral fixation with lens capsule preservation was performed 1 week after the initial surgery. The secondary surgery involved inserting a 30 G needle between the lens capsule and iris, with IOL fixation to the sclera using a double-needle technique. The IOL optics were successfully captured through the incomplete anterior CCC of the preserved lens capsule. No intraoperative vitreous prolapse occurred, eliminating the need for a vitrectomy.

**Outcomes:**

The IOL remained well-fixed without IOL pupillary capture. Additionally, no significant complications such as retinal detachment or vitreous hemorrhage were observed.

**Lessons:**

Preserving the capsule during secondary intrascleral fixation for IOL dislocation in patients with incomplete CCC offers several advantages, including reduced intraoperative vitreous prolapse, minimized surgical invasiveness, suppression of postoperative iris flutter, and prevention of IOL capture within the pupil. However, the long-term outcomes, including the potential risk of lens capsule drop, warrant further investigation with more cases.

## 1. Introduction

Phacoemulsification with intraocular lens (IOL) implantation is the gold standard for cataract surgery. However, achieving stable IOL fixation can be challenging in the case of zonular weakness or incomplete continuous curvilinear capsulorhexis (CCC). When intracapsular fixation fails, alternative techniques such as sulcus or intrascleral fixation should be considered.^[1–4]^

Sulcus fixation,^[5,6]^ while less invasive, is often unsuitable when the anterior capsule or zonules are compromised, leading to suboptimal IOL stability. Furthermore, the proximity of the IOL to the iris increases the risk of complications such as pigment dispersion syndrome,^[6,7]^ uveitis, and secondary glaucoma. By contrast, intrascleral fixation offers reliable IOL stability but often necessitates capsule removal and vitrectomy, increasing invasiveness and surgical complexity and duration. Furthermore, this approach may lead to postoperative iris flutter, potentially causing IOL pupillary capture, reverse pupillary block, an increase in intraocular pressure, uveitis, glaucoma, and anterior chamber hemorrhage syndrome.^[8]^

Although studies^[9,10]^ have demonstrated the feasibility of preserving the capsular bag during intrascleral fixation in single-stage procedures for zonular weakness, evidence supporting its application in secondary procedures remains limited.^[11]^ Aside from our prior report describing favorable outcomes of secondary intrascleral IOL fixation with capsule preservation in aphakic eyes with pseudoexfoliation syndrome (PEX) and zonular dehiscence following phacoemulsification,^[11]^ we are not aware of other similar cases in the literature.

Herein, we report a case of secondary flanged intrascleral IOL fixation with deliberate preservation of the capsular bag in a patient who experienced IOL dislocation after mature cataract surgery complicated by incomplete CCC.

## 2. Case report

The requirement for approval of this case report was waived by the ethics committee at Saitama Red Cross Hospital owing to the retrospective nature of the study. This case report adheres to the Declaration of Helsinki. Written informed consent for publication was obtained.

A 56-year-old Japanese woman was referred for cataract surgery. Her preoperative best-corrected visual acuity (BCVA) was consensus luminescence in the right eye and 1.2 in the left eye. Intraocular pressure (IOP) was 16 and 19 mm Hg in the right and left eyes, respectively. The right eye had disused exotropia and a mature nuclear cataract (grade 5), while the left eye had previous IOL implantation with no retinal or vitreous abnormalities. There was no history of conditions known to cause zonular weakness (e.g., ocular trauma, PEX, atopic dermatitis, or uveitis), and phacodonesis was not observed during the preoperative examination. Fundus examination of the right eye was not possible owing to cataract opacity; however, B-mode ultrasonography ruled out retinal detachment. Corneal endothelial cell density in the right eye was 2154 cells/mm². The patient was otherwise healthy, with no systemic comorbidities such as diabetes mellitus or cardiovascular disease. She did, however, have claustrophobia that required general anesthesia during cataract surgery on the left eye approximately 10 years earlier. There was no known family history of ocular or systemic disease.

Cataract surgery on the right eye, performed using the CENTURION^®^ Vision System (Alcon, TX), revealed weak zonular support. The CCC was initially small but successfully completed. Phacoemulsification and irrigation/aspiration were performed to remove the lens nucleus and cortex, followed by implantation of a 1-piece acrylic IOL (DIB00V, +18.0D; Johnson & Johnson Surgical Vision, Santa Ana, CA). During CCC enlargement, a peripheral tear occurred from the 3 o’clock to 6 o’clock positions, but no vitreous prolapse or IOL dislocation was observed.

**Figure 1.**
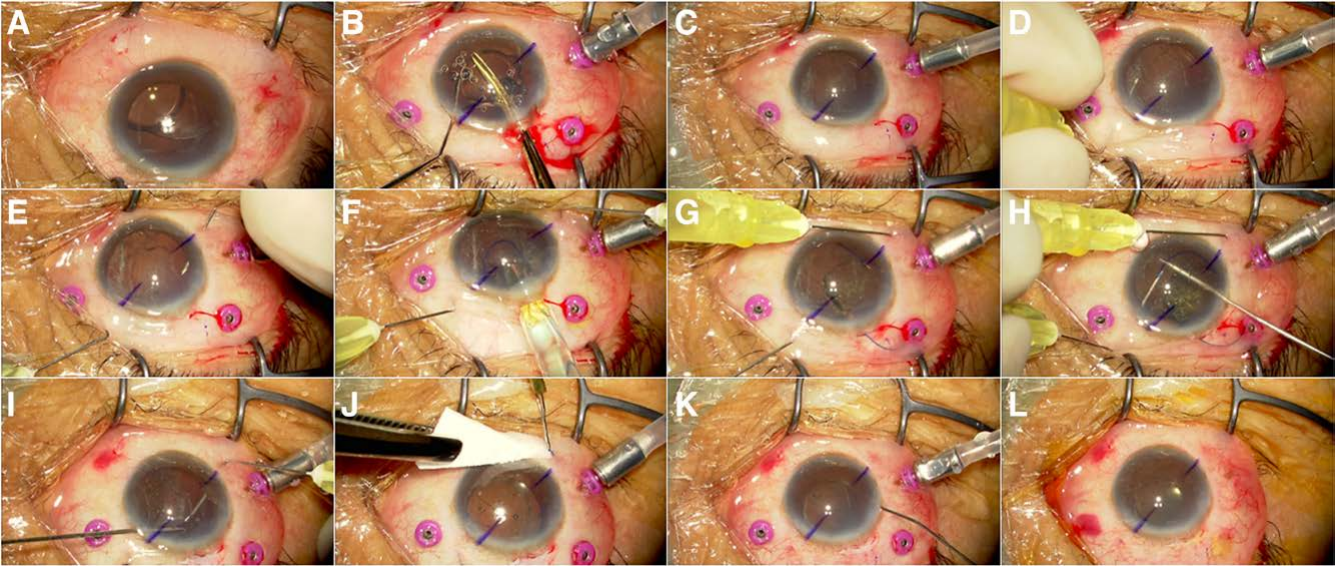
Intraoperative observation during scleral intraocular lens (IOL) fixation. (A) Preoperative findings showing nasal-inferior IOL displacement. (B) Initially, three 27-gauge trocars were inserted in the pars plana in preparation for vitreous prolapse. Subsequently, the dislocated IOL was removed after being bisected. (C) This image displays the situation after IOL removal. (D, E) A 30-gauge needle was inserted at each of the 1 o’clock (D) and 7 o’clock (E) positions, passing between the iris and lens capsule. (F, G) A 3-piece IOL was inserted using an injector. (H–J) The IOL was secured to the sclera using the double-needle technique (H, I) and the flange method (J). (K, L) No vitreous prolapse was observed, and the three 27-gauge trocars were removed; thus, vitrectomy was not performed, and the lens capsule was preserved.

On the first postoperative day, corneal edema was observed; however, no other substantial abnormalities were noted. By the fourth postoperative day, the IOL was found to be dislocated inferiorly toward the nasal quadrant. This displacement suggested an anterior capsule defect and a zonular defect in the region from 3 o’clock to 6 o’clock (Fig. 1A). Seven days after the initial surgery, the patient underwent IOL removal and secondary intrascleral fixation (Fig. 1 and Supplemental Video, Supplemental Digital Content, https://links.lww.com/MD/P258 demonstrating intrascleral IOL fixation with capsule preservation in the present case).

Initially, in preparation for potential vitreous prolapse during the procedure, three 27-gauge vitreous trocars were inserted in the pars plana at the beginning of the surgery. The dislocated IOL was bisected and removed. A 30-gauge needle was inserted 2 mm posterior to the limbus, exiting between the lens capsule and iris, and a 3-piece IOL (NX70S, +17.5D; Santen, Osaka, Japan) was implanted using an injector. The IOL was secured anterior to the lens capsule intrasclerally using the double-needle technique. The IOL optics were captured through the remaining CCC, except on the inferior nasal side. No vitreous prolapse occurred throughout the surgery. Consequently, the vitreous trocars were removed, but vitrectomy was not performed, thereby preserving both the lens capsule and the vitreous body.

One week postoperatively, the BCVA improved to 0.5, with an IOP of 17 mm Hg. Two weeks later, the BCVA improved further to 0.9, with an IOP of 16 mm Hg, and remained stable for 1 month. By 2 months, the BCVA was 1.2, with an IOP of 15 mm Hg. Moreover, the corneal endothelial cell density decreased by 42% to 1242 cells/mm².

**Figure 2.**
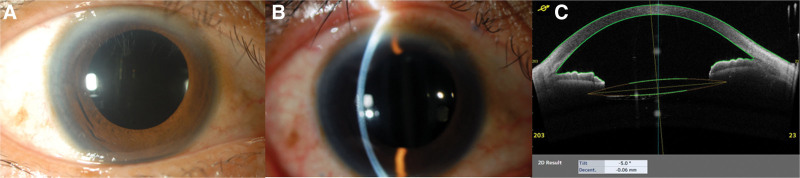
Postoperative findings 3 months after scleral IOL fixation. (A, B) Anterior segment photograph with general illumination (A) and slit-lamp optical section of the anterior segment (B). Anterior segment photographs showing a clear cornea, round pupil, and no iris damage. (C) Anterior optical coherence tomography (OCT) image showing adequate IOL fixation without IOL pupillary capture; IOL tilt is 5.0°, and decentration is 0.06 mm.

Postoperative evaluation revealed a minor IOL tilt (5°) and decentration (0.06 mm), indicating stable fixation (Fig. 2). No major complications such as retinal detachment or vitreous hemorrhage were noted.

Follow-up examinations were conducted on postoperative day 1; at 1 week, 2 weeks, and 1 month; monthly through 3 months; and every 3 months for the first year. At each visit, the patient underwent slit-lamp examination, dilated fundus examination, anterior segment optical coherence tomography, and specular microscopy. Postoperative medications included topical moxifloxacin (4 times daily), 0.1% betamethasone (4 times daily), and bromfenac (twice daily) for the first month. Thereafter, the corticosteroid was switched to 0.1% fluorometholone, which was continued for up to 3 months. At the 3-month follow-up, the preserved capsular bag remained well-positioned, with no signs of inferior displacement, optic decentration, or IOL dislocation. After this period, the patient was referred back to a local ophthalmologist for continued care, and long-term follow-up at our center was not pursued. Nevertheless, considering the preserved capsular bag, long-term surveillance remains essential to detect potential late complications such as capsular fibrosis, contraction, downward displacement, or delayed IOL decentration. Continued follow-up at 6 months, 1 year, and beyond—preferably including anterior segment optical coherence tomography—is recommended to monitor the integrity and positioning of both the capsular bag and IOL.

## 3. Discussion

This report describes secondary intrascleral fixation with lens capsule preservation to address IOL dislocation after mature cataract surgery complicated by peripheral extension of the rhexis during CCC enlargement, resulting in an incomplete CCC with a tear. The procedure utilized a double-needle technique, preserving both the lens capsule and vitreous body without significant complications such as retinal or choroidal detachment or vitreous hemorrhage.^[12,13]^ This case is particularly notable due to the early-onset IOL dislocation in the context of an incomplete CCC and zonular weakness. This constellation of findings represents a distinct and challenging surgical scenario—one that differs from typical cases where sulcus fixation is possible due to sufficient capsular support or from those requiring conventional scleral fixation after complete capsular loss.

Sulcus fixation is commonly performed in cases of capsular rupture.^[5,6]^ However, this technique is not always feasible. A clear contraindication to sulcus IOL implantation is inadequate anterior capsular support.^[6]^ When the anterior capsule is insufficient, alternative fixation methods—such as iris fixation, scleral fixation, or an anterior chamber IOL—must be considered. In this patient, the anterior capsule and zonular support appeared compromised, particularly from 3 o’clock to 6 o’clock, making sulcus fixation precarious. Therefore, we opted for a more reliable scleral fixation.

Various techniques for scleral fixation of IOLs have been described and can be broadly classified into sutured and sutureless approaches. Sutured IOL intrascleral fixation^[14]^ involves creating a scleral flap to bury the suture and requires temporarily externalizing the IOL haptics for suturing, thus complicating the surgical procedure. Additionally, sutures may loosen over time, potentially leading to IOL dislocation over time.^[15]^ To address these limitations, Ye et al^[16]^ proposed a modified Hoffman pocket technique that minimizes conjunctival trauma and provides secure fixation with good visual outcomes, especially in patients with thin sclera.

Sutureless techniques avoid suture-related complications and are generally less invasive. Januschowski et al^[17]^ reported a sutureless approach using the Carlevale IOL system through various vitrectomy port gauges, noting that 27-gauge ports were associated with reduced postoperative hypotony compared to 23-gauge access. Flanged sutureless intrascleral fixation using the double-needle technique, first described by Yamane et al,^[3]^ has gained widespread adoption due to its procedural efficiency and biomechanical stability. This method employs a 30-gauge thin-walled needle to externalize the IOL haptics and create a flanged end through cauterization, allowing for strong fixation with minimal surgical trauma and short operative time. We have frequently employed this technique in clinical practice.^[4,11]^ Accordingly, it was selected for the current case.

Despite its advantages, the Yamane technique is not without risks. Yee et al^[18]^ described a case of cyclodialysis cleft following IOL fixation using this technique, likely due to inadvertent tracking of the haptic through the supraciliary space. This complication underscores the importance of careful needle positioning, particularly in eyes with thin sclera or high axial myopia. These findings suggest that both sutureless and modified sutured techniques offer distinct advantages depending on the clinical scenario and anatomical considerations.

Previous studies^[9,10]^ have demonstrated the feasibility of combining phacoemulsification with scleral fixation of the IOL while preserving the capsule, often using a capsular tension ring and IOL optic capture through the anterior CCC. However, evidence supporting capsular preservation during scleral fixation in “secondary procedures” remains limited.^[11]^ We have reported favorable outcomes in 3 aphakic patients with PEX and zonular dehiscence, showing excellent results with secondary intrascleral IOL fixation and capsule preservation.^[11]^ To our knowledge, no other reports have described capsule-preserving scleral fixation of the IOL as a secondary intervention.

The capsular-preserving technique offers several advantages over conventional methods, which include the following: (1) By leaving the anterior capsule in place, the optics of the IOL can be captured through the anterior CCC, allowing better, more physiological IOL fixation. Regarding complications, preserving the capsule may prevent iris flutter and pupillary capture of the IOL. In the conventional method, the capsule is removed, which can cause iris flutter and make it easier for the IOL to be pupillary captured. IOL pupillary capture, a common complication of scleral fixation, occurs in approximately 8.0%–8.3% of cases.^[3,8,19]^ Preserving the capsule may suppress iris movement, thereby minimizing these complications. (2) The risk of intraoperative IOL drop is reduced. Conversely, with the conventional method, the newly inserted IOL can drop onto the retina during surgery because the lens capsule and vitreous body are removed. (3) The capsule-preserving technique increases the possibility of preserving the vitreous body and avoids unnecessary vitrectomy, making the surgery less invasive and shortening the surgery time. By contrast, the conventional method requires capsular removal and vitrectomy, which makes the procedure more invasive and time-consuming.

Despite its advantages, capsular-preserving techniques pose several challenges. Achieving precise anterior fixation can be technically difficult, and the preserved capsule may obstruct IOL positioning, resulting in tilt or decentration. However, in this case, the IOL was successfully fixed anterior to the capsule without complications such as vitreous prolapse or capsule damage. Furthermore, capturing the optics of the IOL through the anterior CCC of the preserved capsule, as demonstrated in this case and previous studies,^[9,10]^ achieves a more favorable and physiological state of IOL fixation, akin to intracapsular fixation. The observed IOL tilt of 5° and eccentricity of 0.06 mm were within acceptable limits, similar to previously reported ranges (tilt: 3.8°–8.8°; eccentricity: 0.39–0.60 mm).^[3,20–25]^ Excessive IOL tilt (≥7°–10°) or eccentricity (>0.4 mm) has been associated with adverse visual outcomes.^[20,26,27]^ In this case, the IOL alignment was stable, with minimal impact on visual function.

Moreover, during the surgery using the capsular-preserving method, the capsule targeted for preservation could be accidentally caught by 1 of the 2 needles, potentially leading to complications such as vitreous prolapse, zonular rupture, ciliary body hemorrhage, or dissection. Conversely, in the conventional technique, removing the lens capsule and vitreous simplifies the procedure. When inserting the double-needle insertion for IOL fixation in the conventional method, the surgeon needs to be cautious only with the iris, as there is no interference from the lens capsule. However, in the capsular preservation method, by carefully performing the procedure slowly and ensuring sufficient space between the iris and the lens capsule through the insertion of a viscoelastic substance, even a puncture by 1 of the 2 needles can be corrected through careful repuncturing, thus avoiding such complications. However, if vitreous prolapse is observed during surgery using the capsular preservation method, not adhering strictly to this method is advised. Instead, the surgical approach should be promptly switched to pars plana vitrectomy using a wide-angle fundus system, and the conventional method involving the removal of the capsule and vitreous should be promptly adopted without hesitation to avoid severe complications such as retinal detachment and vitreous hemorrhage.

Additionally, with the capsule-preserving method, there is a possibility that the preserved capsule may collapse over the long-term postoperative period. In cases documented here and in previous reports,^[9–11]^ no dropping of the capsule was observed during the postoperative follow-up period of 3 months to 1 year.^[11]^ However, continued long-term monitoring is necessary to check for potential capsule collapse. Furthermore, unlike the conventional method, where removing the capsule eliminates the risk of posterior capsule opacification, this opacification does occur in the capsule-preserving method^[11]^ during the postoperative period.

This study has some limitations. First, as a single-case report, its generalizability is inherently limited. Second, the follow-up period was relatively short (3 months), and long-term outcomes—such as the stability of the preserved capsule or IOL decentration—remain unknown. Further studies involving large cohorts and extended follow-up are warranted to validate these findings.

In conclusion, this case illustrates the feasibility and potential benefits of capsule-preserving flanged intrascleral IOL fixation in a complex clinical setting involving incomplete CCC and suspected zonular weakness following mature cataract surgery. Preserving the residual capsule allowed for secure optic capture and obviated the need for vitrectomy, thereby reducing surgical invasiveness and potentially minimizing postoperative complications such as iris flutter and IOL pupillary capture. This approach may represent a valuable alternative in select cases where sulcus fixation is not feasible and complete capsule loss is absent.

While the short-term outcomes in this case were favorable, the long-term behavior of the preserved capsular bag—such as the risk of collapse, opacification, or IOL decentration—remains uncertain. Further investigation is warranted through large case series with extended follow-up periods to evaluate the long-term safety and stability of this technique. In addition, comparative studies are needed to determine optimal fixation strategies based on the extent of capsular and zonular integrity. Establishing clear selection criteria will be essential for expanding the indications of capsule-preserving IOL fixation in secondary surgical settings.

## Acknowledgments

We thank Editage (www.editage.com) for the English language editing.

## Author contributions

**Conceptualization:** Suguru Nakagawa, Kiyoshi Ishii.

**Data curation:** Suguru Nakagawa.

**Formal analysis:** Suguru Nakagawa.

**Investigation:** Suguru Nakagawa.

**Methodology:** Suguru Nakagawa.

**Supervision:** Kiyoshi Ishii.

**Writing – original draft:** Suguru Nakagawa.

**Writing – review & editing:** Suguru Nakagawa, Kiyoshi Ishii.

## Supplementary Material


